# Emil Wolfgang Menzel, Jr. (1929–2012): Chimpanzee Renaissance Man

**DOI:** 10.1371/journal.pbio.1001384

**Published:** 2012-08-28

**Authors:** William D. Hopkins, David A. Washburn

**Affiliations:** 1 Neuroscience Institute, Department of Psychology and Language Research Center, Georgia State University, Atlanta, Georgia, United States of America; 2 Division of Developmental and Cognitive Neuroscience, Yerkes National Primate Research Center, Atlanta, Georgia, United States of America

## Abstract

William Hopkins and David Washburn pay tribute to a pioneer in primatology and comparative psychology.

**Figure:**
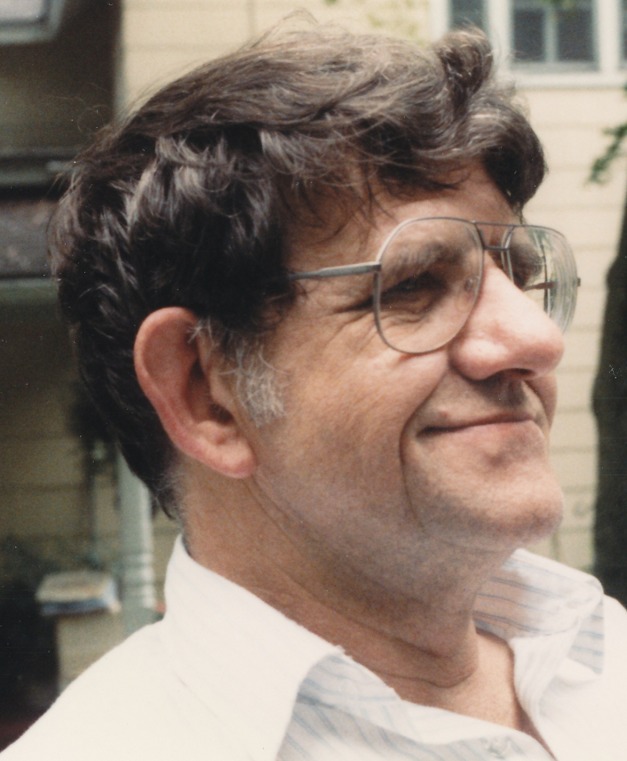
Emil Wolfgang Menzel, Jr.

Emil Wolfgang Menzel, Jr., died April 7th, 2012, and with his passing, the field of psychology and primatology lost a true icon. In many ways, Emil's pioneering observations and research laid the foundation and set the precedent for many contemporary research topics in psychology and primatology including non-verbal and gestural communication, theory-of-mind (before this was the fashionable term), and behavioral economics. His keen eye and critical mind, coupled with his sense of fairness, objectivity, and dry wit will be greatly missed.

Emil Menzel was born in India in 1929 to Ida and Emil Sr. A curious child, Emil's parents, both missionaries, encouraged his interest in natural history, a curiosity that never faded. Returning to the US, Emil completed a BA in English and Philosophy from Elmhurst College and an MA in English from the University of Michigan. After serving 2 years in the Korean War, Emil returned to the United States and completed a PhD in psychology from Vanderbilt University in 1958.

Early in his career, Emil studied a variety of species but his most significant scientific contributions came from his work with chimpanzees. Emil originally worked at the Yerkes Primate Center, with Harry Nissen and Richard Davenport, as their studies examining the effect of different social rearing experiences on social and cognitive development were coming to an end. Emil was perhaps most well known for his work on communication and cognition with a group of chimpanzees living in a one-acre forest. His most famous studies involved experiments in which he would take a single individual in the group out into the forest and show them the location of food or other type of stimulus. After returning the “knower” chimpanzees to their group, he'd release the entire group into the forest. Emil was interested in determining how the “knower” would communicate (intentionally or otherwise) and how they would navigate the forest. On the basis of his keen observations, Emil was able to describe the sophisticated means by which the chimpanzees would learn to follow or use social cues of the “knower” chimpanzee to make inferences about the location of the object or other properties of the stimuli. This work laid the foundation for his seminal papers on cognitive mapping and the representation of space [1],[2].

Another set of landmark studies by Emil involved his descriptions of cooperative tool use in captive chimpanzees [3]. As the story was told to us, while working at the Tulane Primate Center, a number of chimpanzees had learned to escape from their enclosure. Interestingly, the escapes almost always occurred after the researchers and care staff had left for the day, suggesting that the apes were inhibiting their behavior until circumstances were ripe for a break out. To find out what the chimpanzees were doing in their efforts to escape, Emil and his colleagues set up a camera to film their behavior while no one was present. As it turned out, the chimpanzees were dragging long branches to the enclosure wall and holding the branches, like poles, which allowed the chimpanzees to scale the wall and leap over the top. Emil's demonstration of cooperative behavior by the chimpanzees remains at the forefront of current debates over the role of cooperation in the evolution of social cognition and language.

Though a naturalist and ethologist at heart, Emil did not sit idly as technology improved and allowed for alternative ways of testing chimpanzee cognition. He was one of the first scientists to use video technology to ask questions about chimpanzees' understanding spatial relations, particularly in regard to the use of ego- and allocentric cues [4]. As graduate students, we had the pleasure of working directly with Emil on his studies aimed at assessing chimpanzee spatial cognition. Toward that end, in a number of experiments we showed the location of hidden foods to chimpanzees via television monitors and then mapped their travel patterns in locating the foods as well as their use of different landmarks in determining foraging patterns. His attention to detail was meticulous. Before testing, we had to create a faithful pictorial representation of all the potential landmarks and features in the outside enclosure so that we could precisely determine their travel and foraging patterns. One day we enlarged a pictorial representation of the chimpanzees' outside enclosure and simply pointed to the baited location in the enclosure on the map to see whether the apes would then immediately travel to that location (which they were able to do). Similarly, in the late 1980s, when the joystick computer system for testing learning and cognition in monkeys and apes was developed at the Language Research Center [5], Emil embraced this technology and developed his own tests [6]. We remember very vividly testing the chimpanzees on his tasks. Two things were particularly noteworthy about Emil's computer-based tests: First, they tended to be computerized versions of the field experiments that he or others in the discipline had conducted with nonhuman primates. For example, he developed computerized versions of the barrier problems used by D.O. Hebb early in his career [7]. In this way, Emil's data were particularly valuable for grounding the new computer-task paradigm—in which animals were often performing tasks and demonstrating competencies never before documented in their species—in the rich, existing literature on animal foraging, way-finding, and learning. The second memorable aspect of this testing is that Emil did his own computer programming, both of the actual tests that would be administered to the nonhuman primates and also of the data-analysis software that would simulate, often in real-time graphic representations on the screen, the various potential mechanisms that might explain the animals' behavior. These analyses directly inspired our own approach to data analysis [8]. We fondly recall Emil's excitement in describing these computer-based algorithms and also the enthusiasm with which he discussed his data and the ways that animals' performance on computerized versions of the tasks resembled the behavior of various animals (“…that's just like a chicken…”) in earlier naturalistic, field studies.

Emil Menzel was a very productive scholar, but the scholar we knew was not motivated by amassing more publications. He remains one of the most highly respected researchers in primatology, but he seemed unconcerned about establishing a legacy associated with his name, other than the most important one he established through his children and their children. Indeed, one of our favorite quotes from Emil's writings took a playful jab at the tendency to name apparatus and paradigms after the scientist who developed or popularized them: “In the hope that I can make field work scientifically respectable, I have considered patenting the tree as the Menzel Jumping Stand, the river as the Tulane Obstruction Apparatus, and the jungle as the Delta Primate Center General Test Apparatus” (p. 80, [9]). Rather, Emil was motivated by the data, and by the joys associated with the scholarly pursuit of knowledge about species in which he found fascination. His contributions to the literature and to the many students, colleagues, and collaborators who were influenced by him are tangible reminders of the twinkle that would flash in Emil's eyes whenever he started talking about science. He remained a curious child throughout his lifetime.
